# Author Correction: Toll like receptor 4 mediates cell death in a mouse MPTP model of Parkinson disease

**DOI:** 10.1038/s41598-022-26917-1

**Published:** 2023-01-16

**Authors:** Carmen Noelker, Lydie Morel, Thomas Lescot, Anke Osterloh, Daniel Alvarez-Fischer, Minka Breloer, Carmen Henze, Candan Depboylu, Delphine Skrzydelski, Patrick P. Michel, Richard C. Dodel, Lixia Lu, Etienne C. Hirsch, Stéphane Hunot, Andreas Hartmann

**Affiliations:** 1 INSERM UMR_S975, Université Pierre et Marie Curie Paris 06 UMR_S975, CNRS UMR 7225, CR-ICM, Groupe Hospitalier Pitié-Salpêtrière, 75013 Paris, France; 2 Department of Neurology, Philipps-University Marburg, 35043 Marburg, Germany; 3 Bernhard Nocht Institute for Tropical Medicine, 20324 Hamburg, Germany

Correction to: *Scientific Reports* 10.1038/srep01393, published online 6 March 2013

This Article contains errors in Figures 1 and 2.

In Figure 1C, the two top panels and the two bottom panels are reversed. Additionally, the panel labels contain errors, where the left top panel label should read “TLR-deficient Control”, the right top panel label should read “TLR-deficient MPTP” and the left bottom panel label should read “WT Control”.

In Figure 2C, the panel labels contain errors, where the left top panel label should read “WT Control”, the left bottom panel label should read “TLR-deficient Control” and the right bottom panel label should read “TLR-deficient MPTP”.

The correct Figures 1 and 2 and accompanying legends appear below.

**Figure 1:**
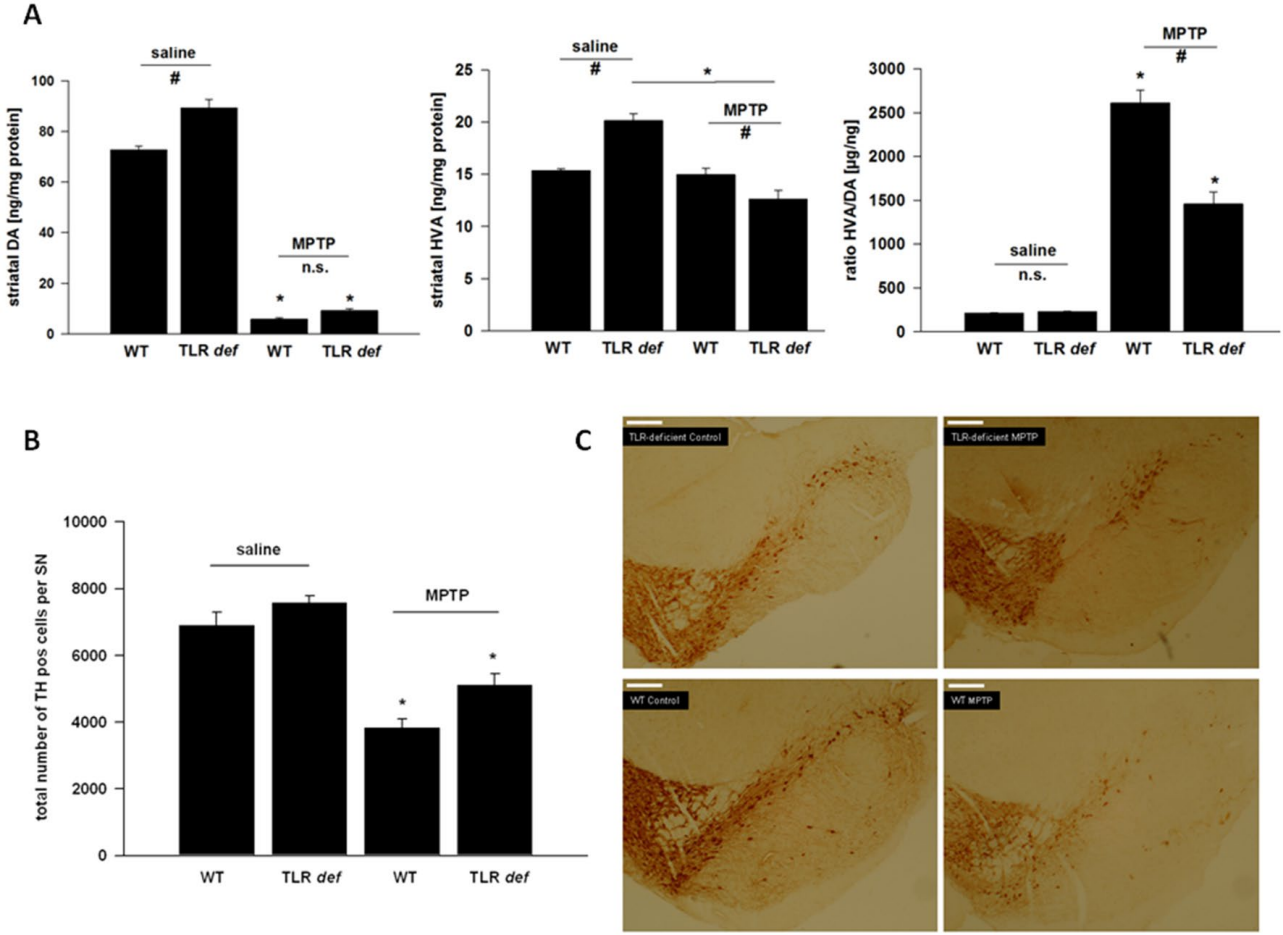
(**A**): HPLC measurement of striatal dopamine (DA) and homovanillic acid (HVA) levels, as well as calculation of the HVA/DA-ratio after MPTP-intoxication of TLR4- deficient (TLR def) and WT mice in comparison to saline treated mice. Values are expressed as mean ± SEM (*p < 0.05 comparing MPTP treated group with its saline control, ^#^p < 0.05 comparing MPTP-treated groups of WT and TLR4-deficient mice. (**B**): TH-positive cell number in SN after MPTP treatment. WT = WT mice, TLR deficient = TLR4 deficient mice. Values are expressed as mean ± SEM (*p < 0.05 comparing MPTP treated group with its saline control). (**C**): Representative photographs of the SN of each treatment group is shown. TH-staining of free-floating cryomicrotome-cut sections at day 7. TLR4-deficient = TLR4-deficient mice, WT = wild-type mice. Control = saline treated mice, MPTP = MPTP-treated mice, Scale bars = 200 μm.

**Figure 2:**
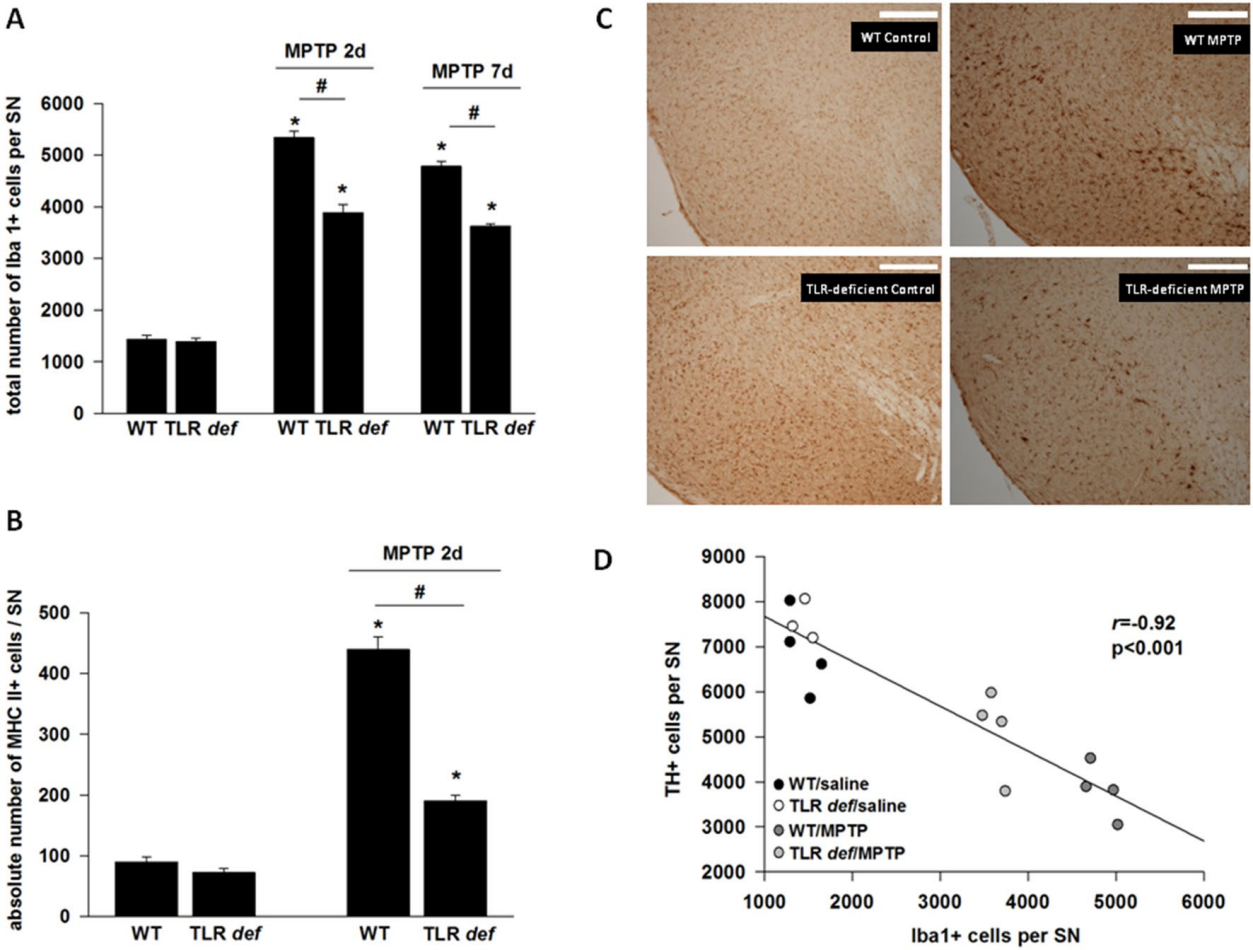
(**A**): Number of nigral Iba+ microglia at day 2 and day 7. WT = WT mice, TLR def = TLR4-deficient mice. Values are expressed as mean ± SEM (*p < 0.05 comparing MPTP-treated group with its saline control, ^#^p < 0.05 comparing MPTP-treated groups of WT and TLR4-deficient mice). (**B**): MHC II+ cell number in SN after MPTP treatment at day 2. WT = WT mice, TLR def = TLR4-deficient mice. Values are expressed as mean ± SEM (*p < 0.05 comparing MPTP-treated group with its saline control, ^#^p < 0.05 comparing MPTP-treated groups of WT and TLR4-deficient mice). (**C**): Representative photographs of the SN (Iba-1 staining) of each treatment group at day 2 are shown. TLR4 def = TLR4-deficient mice, WT = wild-type mice. Control = saline treated mice, MPTP = MPTP treated mice. Scale bars = 200 μm. (**D**): Correlation analysis of TH+- and Iba+-positive cells counts at day 7 in SNpc (r = −0.92, p < 0.001).

